# Righthandedness and Lefthandedness, with Chapters Treating of the Writing Posture, the Rule of the Road, Etc.

**Published:** 1909-05

**Authors:** 


**Righthandedness and lefthandedness,** with Chapters treating of the writing posture, the rule of the road, etc. By **George M. Gould,** M.D. Duodecimo, pp. 210. Illustrated. Philadelphia and London: J. B. Lippincott Co. (Cloth, $1.25.)

If the title of this book does not appeal to one the opening paragraphs will and interest will not fag until the last page is read. It is a study of righthandedness and lefthandedness and is unique in many respects because it tears to tatters some of the mythical beliefs regarding ambidextry.

It is doubtful if many have given much consideration to the question of which hand is the more useful. As a matter of fact both hands have their particular uses, and it is also a fact that some left-handed people are especially expert; yet it is true that a left-handed person cannot in most cases learn to play the piano with any degree of skill or ease. No one has ever seen an Indian pull a bow with his left hand; the puzzling "south-paw" pitcher in a base ball game is puzzling to the batter because his delivery is unusual; not because he is a better pitcher than his right-handed partner; just as the left-handed batter is puzzling to the right-handed pitcher. On the other hand, place a left-handed batsman before a left-handed pitcher and there isn't the slightest confusion on either side. They meet on equal ground. And yet, in the right-handed person the left hand does much more difficult and delicate work, and this Gould explains by discarding any consideration of right or left-handedness and declaring it merely a matter of right eyedness. Which, when it is considered, is quite plausible as an explanation. With the flute and fife, where the hands are not seen by the player, it is the right hand which does the work; so too, with the instruments in the central line of vision as the flaglot for instance.

There is one startling statement in the book and it is that the "ambidexterity crank is deserving of more severe punishment than any other of our many criminal insane;" another is that the 20,000 000 lateral spinal curvatures are due wholly to products of morbid visual function.

The illustrations are excellent and clearly descriptive, and the chapters which appeal to one's interest most forcibly are the origin of righthandedness; study of a case of two-handed synchronous writing; and the pathologic results of righteyedness and lefteyedness.

The book is an excellent one in every respect.

